# Reappraisal and Distraction Emotion Regulation Strategies Are Associated with Distinct Patterns of Visual Attention and Differing Levels of Cognitive Demand

**DOI:** 10.1371/journal.pone.0162290

**Published:** 2016-11-17

**Authors:** Gregory P. Strauss, Kathryn L. Ossenfort, Kayla M. Whearty

**Affiliations:** State University of New York at Binghamton, Department of Psychology, Binghamton, NY 13902, United States of America; Tilburg University, NETHERLANDS

## Abstract

Multiple emotion regulation strategies have been identified and found to differ in their effectiveness at decreasing negative emotions. One reason for this might be that individual strategies are associated with differing levels of cognitive demand and require distinct patterns of visual attention to achieve their effects. In the current study, we tested this hypothesis in a sample of psychiatrically healthy participants (n = 25) who attempted to down-regulate negative emotion to photographs from the International Affective Picture System using cognitive reappraisal or distraction. Eye movements, pupil dilation, and subjective reports of negative emotionality were obtained for reappraisal, distraction, unpleasant passive viewing, and neutral passive viewing conditions. Behavioral results indicated that reappraisal and distraction successfully decreased self-reported negative affect relative to unpleasant passive viewing. Successful down regulation of negative affect was associated with different patterns of visual attention across regulation strategies. During reappraisal, there was an initial increase in dwell time to arousing scene regions and a subsequent shift away from these regions during later portions of the trial, whereas distraction was associated with reduced total dwell time to arousing interest areas throughout the entire stimulus presentation. Pupil dilation was greater for reappraisal than distraction or unpleasant passive viewing, suggesting that reappraisal may recruit more effortful cognitive control processes. Furthermore, greater decreases in self-reported negative emotion were associated with a lower proportion of dwell time within arousing areas of interest. These findings suggest that different emotion regulation strategies necessitate different patterns of visual attention to be effective and that individual differences in visual attention predict the extent to which individuals can successfully decrease negative emotion using reappraisal and distraction.

## Introduction

Humans experience a variety of negative and positive emotions that arise in response to internally generated mental representations or external cues. It is often adaptive to regulate these emotions in certain contexts (i.e., to apply strategies to increase or decrease positive or negative affect). Multiple theoretical frameworks have been developed to explain the complex interplay between emotion generation and regulation (for a review see [1]). In what is perhaps the most influential theoretical framework, the process model, Gross [2, 3] proposes that emotions unfold over a multi-componential sequence that involves four stages: stimulus, attention, appraisal, and response [3]. In the initial stage, individuals perceive a *stimulus* within its current context. Stimuli can consist of external cues or internally generated mental representations. Second, an individual must *attend* to a stimulus or its attributes, causing stimuli subjected to selective attention to be passed along for additional cognitive processing where they are gated into working memory and subjected to more elaborative processing. Unattended stimuli may not reach threshold for elaborative processing, and are therefore less likely to be carried forward to later stages of the emotion generation process. Third, individuals *appraise* a stimulus and evaluate its valence (i.e., pleasant or unpleasant) and relevance for current goal states. Finally, stimuli that are appraised give rise to *responses* at the subjective (i.e., experiential), objective (i.e., outward expressivity), and physiological levels. Importantly, at any one of these stages, strategies can be applied to control the intensity, duration, or frequency of positive and negative emotions. The application of strategies to control our emotions can occur outside of conscious awareness (i.e., implicitly) [4] however, the majority of studies to date have examined the effects of explicit goals on altering the 4 different stages of the emotion generation sequence [5]

Emotion regulation strategies are generally divided into those that are antecedent-focused or response-focused [2]. Antecedent-focused strategies are those that are applied before the emotion generation process has fully completed (e.g., reappraisal, distraction, attentional deployment), while response focused strategies encompass attempts to modulate affect after an emotional experience, behavior, or physiological response has occurred (e.g., suppression) [2, 3]. There is some evidence that effective implementation of antecedent and response focused strategies relies on common underlying neural circuitry, where prefrontal and cingulate cortices support cognitive control processes that modulate activation of subcortical and posterior circuits involved with emotion generation [6, 7]. However, not all emotion regulation strategies are equally effective at reducing the response to unpleasant stimuli. Differences have been demonstrated between antecedent and response-focused strategies, such that antecedent focused strategies have been shown to reduce the subjective and physiological response to unpleasant stimuli, whereas response focused strategies increase sympathetic activation [2]. There are also large individual differences in how effectively participants can apply different strategies to control emotional response. However, relatively few studies have attempted to identify cognitive processes that predict individual differences in how effectively individuals apply different strategies or why some strategies are more effective than others.

One possible explanation for differential effectiveness among strategies and individual differences in emotion regulation success is that some strategies may require distinct patterns of visual attention to achieve their regulatory effects and that participants may have important individual differences in emotion-attention interactions when applying these strategies. To evaluate this possibility, a study by van Reekum et al. [8] recorded eye tracking, pupil dilation, and functional neuroimaging data concurrently during a reappraisal paradigm that required participants to either increase, decrease, or passively view unpleasant stimuli. They found that decreasing negative affect via reappraisal resulted in fewer total fixations and less time fixating on emotional areas of interest within the image relative to passively viewing unpleasant stimuli. Subsequent eye tracking studies have confirmed these results, indicating that the decreasing negative affect via reappraisal or suppression results in reduced dwell time to arousing interest areas compared to an unpleasant passive viewing condition [9, 10]. However, it is unclear whether individual differences in reducing emotional response to unpleasant stimuli are associated with decreased attention to arousing interest areas or increased attention to arousing interest areas. For example, van Reekum et al. [8] found that greater success at decreasing negative emotion via reappraisal, as indexed by decreased amygdala activation, was associated with a reduced number of fixations in arousing interest areas. Similarly, Manera et al. [10] found that proportion of dwell time to arousing areas of social stimuli mediated the effects of up- and down-regulation instruction on subjective emotional experience during reappraisal (i.e., increase instructions resulted in greater attention to arousing interest areas relative to unpleasant passive viewing, whereas decrease instructions resulted in less attention to arousing regions than unpleasant passive viewing). In contrast, Bebko et al. [9] found that greater self-reported decreases in negative affect during reappraisal and suppression were associated with *increased* duration of attention to the most arousing scene regions. Thus, it is currently unclear how visual attention plays a role in individual differences in emotion regulation success, as well as whether different strategies achieve their regulatory effects via distinct patterns of visual attention.

Another potential explanation for the differential effectiveness of individual emotion regulation strategies and individual differences in emotion regulation success is that some strategies are more cognitively demanding than others and participants differ in their capacity or willingness to engage in cognitively effortful emotion regulation processes. Pupil dilation has long been used as a marker of emotional arousal and sympathetic nervous system activity, with greater pupil dilation reflecting increased emotional reactivity [11]. However, more recent studies have demonstrated that pupil response is also sensitive to effort and level of cognitive demand [12, 13], as well as changes in cognitive control dynamics [14–16]. Emotion regulation studies have used pupil dilation as a marker of emotional reactivity, as well as the recruitment of effortful cognitive control processes. Several studies have indicated that both increasing and decreasing negative emotion via reappraisal results in significant increases in pupil dilation relative to an unpleasant passive viewing condition [8, 17]. These findings suggest that pupil dilation is sensitive to increases in cognitive control above and beyond sympathetic nervous system changes resulting from emotional reactivity. Furthermore, Urry et al. [17] found that greater increases in pupil dilation during a decrease reappraisal condition predicted reduced amygdala activation and greater increases in pupil dilation during an increase reappraisal instruction predicted greater amygdala activation. However, not all studies have found reappraisal to result in greater pupil dilation than unpleasant stimulus passive viewing conditions, or that greater recruitment of effortful cognitive processes results in better emotion regulation. For example, Bebko et al. [9] found that pupil size was significantly smaller for both reappraisal and suppression than an unpleasant stimulus passive viewing condition, and that reduced pupil dilation was associated with better emotion regulation success. Thus, it is unclear whether various emotion regulation strategies are associated with differing levels of cognitive demand, and whether individual differences in emotion regulation success are predicted by the extent to which participants recruit effortful cognitive control processes.

The current study aimed to determine whether: 1) different emotion regulation strategies are associated with distinct patterns of visual attention and differing levels of cognitive demand, and 2) individual differences in emotion regulation success are predicted by visual attention and the recruitment of effortful cognitive control processes. Participants were asked to view unpleasant or neutral stimuli while eye movements and pupil dilation were recorded. Instructions required participants to either passively view unpleasant stimuli or decrease negative emotion using two emotion regulation strategies: reappraisal and distraction. In the literature on emotion regulation, reappraisal instructions generally take two forms, reinterpretation or distancing. The reinterpretation form of reappraisal requires participants to generate an alternate meaning for the visual stimulus in an effort to feel more neutral. This variant of reappraisal was selected because it is widely used in the literature and thought to be highly cognitively demanding, involving several cognitive processes, including directing attention to reappraisal relevant stimulus features, holding reappraisal goals and the content of the initial appraisal in working memory, inhibiting the prepotent appraisal, and generating a goal-relevant reappraisal by drawing on semantic memory. The role of visual attention during the reinterpretation form of reappraisal is unclear because the majority of prior studies have either used distancing forms of reappraisal or allowed participants to freely choose between reinterpretation or distancing on each trial [8, 10, 17]; given that most individuals tend to select distancing when given a choice between the two reappraisal tactics [18], the role of visual attention during the reinterpretation form of reappraisal is unclear. To determine whether visual attention and effortful cognitive control processes differ across emotion regulation strategies, distraction was selected as a comparison condition. Distraction involves trying to feel more neutral by generating a mental representation of something unrelated to the presented stimulus. In a neuroimaging study, McRae et al. [19] found that distraction and reappraisal had both similarities and differences in patterns of neural activation. Specifically, both strategies decreased amygdala response and resulted in increased activation in prefrontal and cingulate corticies. However, compared to reappraisal, distraction led to greater decreases in the amygdala and greater increases in prefrontal and parietal regions. One potential explanation for the neurophysiological findings of McRae et al. [19] is that the two strategies impose different demands on attention or that they occur at different stages of attention and the emotion generation sequence. A study that used Event Related Potentials (ERP) to directly compare reappraisal and distraction shed light onto this question, indicating that distraction achieves its regulatory effects at an earlier stage than reappraisal. Specifically, Thiruchselvam et al. [20] found that distraction decreased amplitude of the late positive potential (LPP) at a magnitude that was comparable to reappraisal; however, the reduction in LPP amplitude occurred earlier in time for distraction. It is unclear whether the differences in temporal dynamics that were observed between reappraisal and distraction in Thiruchselvam et al. [20] reflect differences in visual attention between these two conditions. It is plausible that distraction achieves an earlier effect because participants are more rapidly able to attend to less arousing scene regions than when applying reappraisal, which likely requires initial attentional orientation to arousing interest areas and then subsequent disengagement of attention and a shift toward less arousing scene regions at the point during which reinterpretation of the stimulus occurs. Alternatively, distraction may be a less cognitively effortful process than reappraisal, allowing it to be implemented more quickly due to reduced demands. Eye tracking and pupil dilation were recorded during the current study to test these possibilities.

We hypothesized that effective implementation of reappraisal and distraction would necessitate distinct patterns of visual attention. Specifically, consistent with van Reekum et al. [8] and Urry et al. [17], we predicted that decreasing negative affect via reappraisal and distraction would be associated with reduced visual attention to arousing interest areas. However, we expected that distraction would result in decreased attention to arousing interest areas earlier in time than reappraisal. Additionally, we hypothesized that reappraisal and distraction would result in increased pupil dilation relative to an unpleasant stimulus passive viewing condition, suggesting that both strategies rely on effortful cognitive control processes; however, reappraisal was predicted to result in greater pupil dilation than distraction due to the high level of cognitive resources needed to perform the reinterpretation form of reappraisal. Finally, based on prior studies [8, 17], we hypothesized that decreased proportion of dwell time to arousing interest areas and increased pupil dilation would be associated with better emotion regulation success for both reappraisal and distraction conditions.

## Methods and Materials

### Participants

Twenty-five undergraduate students (16 women, 9 men) completed the primary study. Participants were 19.8 years of age on average and 60% were Caucasian, 4% African-American, 4% Hispanic, and 16% Asian. Individuals were excluded from the study if they were less than 18 years old, had lifetime history of psychiatric or neurological diagnoses, were taking psychotropic medications, or had corrected-to-normal vision > 20/50. No participants discontinued participation once experimental procedures had begun. All participants provided written informed consent and received course credit for a study approved by the Binghamton University Institutional Review Board that was conducted in accordance with principles from the declaration of Helsinki.

### Procedure

After completing informed consent, the experimenter explained the concept of emotion regulation and participants were provided with a detailed explanation of how to use reappraisal and distraction to reduce negative affect. Participants were then guided through a series of training trials for each strategy, where feedback and shaping was provided by the experimenter. During training trials, participants were instructed to implement the strategy only after the image appeared on the screen. A series of practice trials were then completed (6 reappraisal, 6 distraction, 4 unpleasant passive viewing, 4 neutral passive viewing), where the experimenter asked participants to verbalize the exact manner in which they used reappraisal or distraction to decrease negative emotion while viewing unpleasant photographs. This procedure was used to ensure that participants understood the instructions and were able to adequately implement each strategy. The experimenter then proceeded with eye tracker setup, and a 9-point calibration and validation was performed prior to administering the emotion regulation task.

### Apparatus

Eye position was recorded monocularly from the right eye at 2000 Hz using an SR Research Eyelink 1000 desk-mounted system. A 9-point calibration was used and drift-correction was performed prior to each trial. Participants were seated 70 cm from a 19” LCD monitor (Dell model P190S) operating at a refresh rate of 60 Hz, with head positioned in a chin-and-forehead rest to reduce motion artifacts.

The Eyelink 1000 was also used to collect pupil data. Pupil activity was examined in Eyelink’s scaled pupil diameter values rather than absolute sizes or percent change from baseline fixation. Scaled values generally ranged between 1000 to 3000, which corresponds to approximately to 1–3 mm [21].

### Stimuli

A total of 112 images from the International Affective Picture System (IAPS) library were used (28 neutral, 84 negative) for the experimental trials. Unpleasant and neutral images significantly differed on normative IAPS valence (F = 196.98, p < 0.001; Unpleasant: M = 2.37, SD = 0.65; Neutral: M = 5.18, SD = 0.69) and arousal ratings (F = 226.23, p < 0.001; Unpleasant: M = 5.98, SD = 0.78; Neutral M = 3.21, SD = 0.71). Images used in the reappraise, distract, and unpleasant passive viewing conditions did not differ on arousal (F = 0.02, p = 0.98; Distract: M = 5.67; SD = 0.85; Reappraise: M = 5.83; SD = 0.95; Unpleasant Passive: M = 5.97, SD = 0.66) or valence (F = 0.06, p = 0.94; Distract: M = 2.38; SD = 0.76; Reappraise: M = 2.53; SD = 0.94; Unpleasant Passive: M = 2.35 SD = 0.65). A different set of 16 unpleasant and 4 neutral images were used during practice trials. Individual IAPS images used in each condition are listed in the Appendix.

### Emotion Regulation Task

The experimental task included 112 trials that were divided into 2 blocks of 56 trials each. There were four trial types: Watch (unpleasant passive viewing), View (neutral passive viewing), Distract, and Reappraise. Each block contained 14 Watch and 14 View trials, as well as either 28 Distract or 28 Reappraise trials. Reappraise and distract conditions were presented in separate blocks to reduce task demands and decrease the likelihood of participants either confusing or combining strategies. Block order was counterbalanced and the order of trials within each block was randomized.

On View and Watch trials, participants were instructed to look at the pictures and allow their thoughts and emotions to unfold naturally. View and Watch were thus similar in that they both involved passive viewing. Different cues were selected for these conditions to ensure that participants had comparable anticipatory knowledge about the upcoming image across the four conditions. On Reappraise trials, participants were instructed to feel neutral in response to the unpleasant image by reinterpreting its meaning to be more neutral. For example, if the image displayed a group of women crying outside of a church, the participant could alter their interpretation of the image by thinking that the women were crying tears of joy after attending a wedding. On Distract trials, participants were instructed to feel neutral in response to the unpleasant image by thinking of something unrelated to the image on the screen, such as a complex geometric shape or common scenes in their neighborhood.

**Fig 1:**
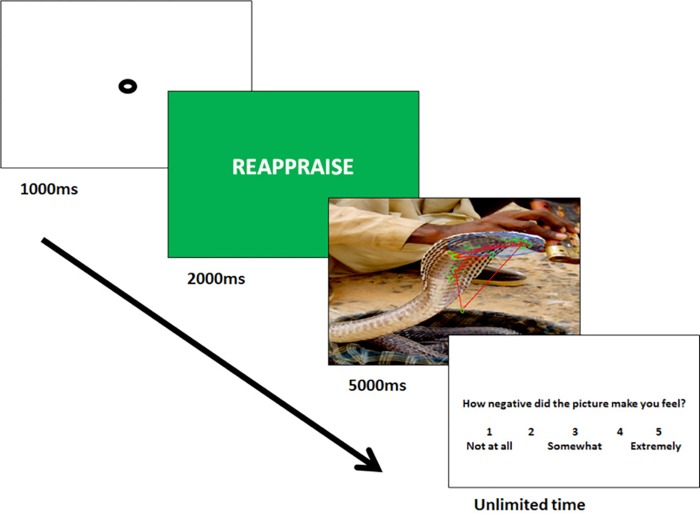
Example Trial Sequence with Eye Tracking Data and Emotional Interest Areas Depicted. The trial started with a 1000ms fixation, followed by a 200ms instruction cue (distract, reappraise, watch, view), a 5000ms IAPS stimulus presentation, and unlimited time for self-reported negative affect. The unpleasant image presented is a sample stimulus. It is not part of the IAPS library and was not included in the task. Actual IAPS images could not be displayed due to copyright limitations.

A sample trial sequence is presented in Fig 1. Each trial started with a central fixation target upon which the participant was required to fixate for 1000ms. This was followed by an instruction cue (View, Watch, Distract, or Reappraise) that was presented in white text on a colored background for 2000ms. Background colors included Blue (Distract), Green (Reappraise), Gray (Watch), and Black (View). The IAPS image was then presented for 5 seconds during which time eye movements and pupil dilation were recorded. Images subtended 33.02 x 20.64 degrees of visual angle. After the image disappeared, participants were prompted to report how negative they felt on that trial using a 1 (not at all) to 5 (extremely negative) scale; unlimited time was provided for self-report.

### Emotional Areas of Interest Delineation

Two follow-up studies were conducted to identify and validate emotional areas of interest (E-AOIs) for the 112 unpleasant IAPS images used in the emotion regulation task. Procedures were modeled after Bebko et al [9]. Follow-up study 1 included a sample of 12 undergraduate students who had normal or corrected-to-normal vision and no history of psychiatric or neurological illness who participated for course credit. These participants performed two tasks. First, they passively viewed all 84 unpleasant IAPS images that were used in the primary experiment while eye movements were recorded. Each image was presented for 3 seconds, subtending 33.02 x 20.64 degrees of visual angle. After viewing each image naturally, participants indicated how negative they felt on that trial using a 1 (not at all) to 5 (extremely) scale. A second task was then completed, where participants were presented with the same series of 84 images within a Microsoft Powerpoint presentation, and given unlimited time to use free-form shapes to cover arousing areas of the scene. Participants were instructed to use the smallest and most concise shapes possible to cover areas of the scene until they no longer felt negative when viewing the image.

E-AOIs were created by combining the results of the two tasks used in follow-up study 1. First, participant eye tracking data obtained in the passive viewing task was aggregated for each image by creating a two-dimensional heat map that reflected mean fixation count and dwell time across each pixel. Second, data resulting from the Powerpoint task was aggregated to form a two-dimensional matrix for each image that reflected the average positioning of free-form shapes across each pixel. Finally, heat maps resulting from the fixation data and two-dimensional matrixes resulting from the Powerpoint task were combined to delineate an E-AOI for each image. Successive clusters of pixels that were identified as negative by at least 50% of participants which were also frequently attended to in the aggregate eye-tracking heat map were selected as EAOIs. This ensured that E-AOIs encompassed commonly viewed scene regions that were also experienced as being negatively valenced by the majority of participants. Two separate members of the research team then evaluated the E-AOI identified for each stimulus to verify that it did indeed capture the most arousing scene regions. No significant discrepancies were noted and the raters reached agreement.

A second follow-up study was conducted to validate the E-AOIs identified in follow-up study 1. A separate sample of 12 undergraduate students who reported no history of psychiatric or neurological diagnosis completed an emotional experience rating task. The 84 unpleasant IAPS images used in the emotion regulation task were presented in random order with the E-AOIs overlaid onto each image as black forms, occluding arousing aspects of the scene. Participants viewed each image for 3 seconds and then reported how negative they felt on that trial using a 1 (not at all) to 5 (extremely negative) scale. Results indicated that participants reported significantly less negative emotion when rating the occluded E-AOI images compared to the full images presented in follow-up study 1 (Full image: M = 3.69, SD = 0.67; E-AOI Occluded Images: M = 2.91, SD = 0.71; F (1, 11) = 6.75, p < 0.05). Thus, follow-up study 2 validated that the EAOIs were identifying highly aversive scene regions.

### Data Analysis

Two sets of analyses that used different interest area sets were conducted to evaluate eye tracking data across conditions. The first set of analyses included data from the neutral, unpleasant passive viewing, reappraise, and distract conditions. The interest area for each stimulus was defined as the image border, allowing for a comparison of the number of fixations and average pupil dilation in relation to the entire image. Separate repeated measures ANOVAs were used to evaluate differences among conditions in total number of fixations and total pupil dilation. These analyses allowed us to evaluate the effects of arousal (i.e., unpleasant vs neutral) and regulation on visual attention and sympathetic nervous system activity (i.e., pupil dilation).

The second set of analyses involved only the conditions displaying aversive content (i.e., reappraise, distract, unpleasant passive viewing). Interest areas were unique for each stimulus and consisted of the E-AOIs developed and validated in the two follow-up studies. Results were segmented into five 1-second epochs to evaluate differences in the time course of visual attention and pupil response across conditions. Repeated measures ANOVAs evaluated differences among conditions on two primary eye tracking dependent variables- total dwell time (i.e., total time in ms for all fixations within an E-AOI) and average pupil dilation at fixation within the E-AOI. As is typical, the first fixation was excluded from analyses of individual trials because it is a byproduct of fixating on the fixation point preceding each trial.

## Results

### Self-Report Analyses

**Fig 2:**
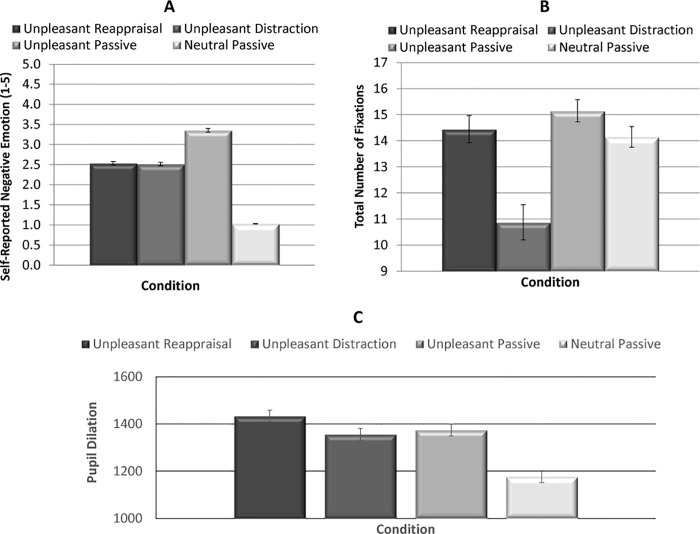
Mean self-reported negative emotion, total fixations, and pupil dilation across the entire image. A) Mean (SE) self-reported negative affect for each condition; B) Mean (SE) total number of fixations throughout the entire image for each condition; C) Mean (SE) pupil dilation hroughout the entire image for each condition.

See S1 Data for corresponding dataset that analyses were performed from. Repeated measures ANOVA revealed a significant within-subjects effect of Condition, F (3, 53.9) = 101.9, p < 0.001 (partial eta squared = 0.81). Follow-up paired samples t-tests indicated that participants reported significantly more negative emotion for unpleasant passive viewing, reappraisal, and distraction trials than neutral trials (p’s < 0.001). Additionally, participants reported significantly less negative emotion for reappraisal and distraction than unpleasant passive viewing trials (p’s < 0.001), indicating that these conditions were effective at down-regulating negative emotion. However, reappraisal and distraction did not significantly differ, suggesting that the two regulation conditions were similarly effective at decreasing negative emotion (t = 0.17, p = 0.87) (see Fig 2A).

### Eye Tracking Fixation Analyses

#### Fixations Across the Entire Image

To examine global differences in visual attention across conditions, fixation count was calculated collapsing across the entire 5 second period, with the image border serving as the AOI. Repeated measures ANOVA indicated a significant difference in the number of total fixations allocated across the conditions, F (3, 50.3) = 27.5, p < 0.001 (partial eta squared = 0.53). Paired-samples t-tests indicated that participants made significantly fewer fixations during distraction than unpleasant passive viewing, neutral passive viewing, or reappraisal (p’s < 0.001). There were a greater number of fixations during unpleasant passive viewing than neutral passive viewing (t = 4.39, p < 0.001). However, the total number of fixations did not differ between reappraisal and either unpleasant passive viewing (t = 1.45, p = 0.16) or neutral (t = 0.64, p = 0.53) conditions (see Fig 2B).

#### Percentage Dwell Time within Emotional Areas of Interest

**Fig 3:**
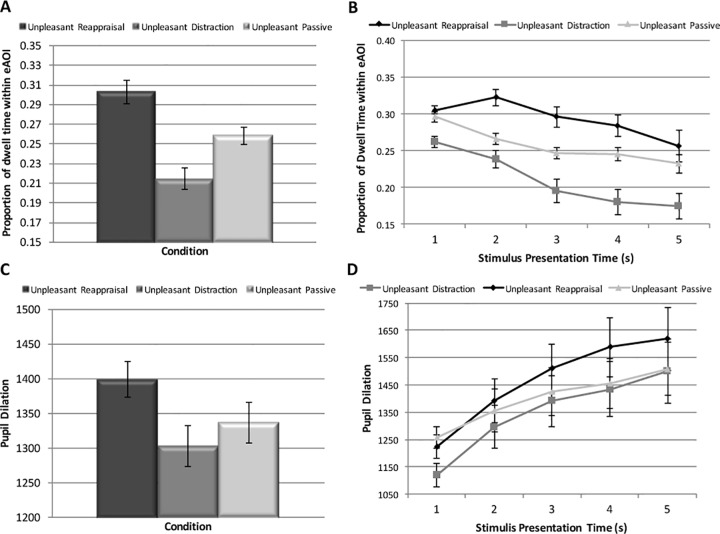
Mean Proportion Dwell Time and Pupil Dilation within the Emotional Areas of Interest. A) Mean (SE) proportion dwell time to the emotional interest areas for each condition, collapsing across the entire 5 second period; B) Mean (SE) proportion dwell time to the emotional interest areas for each condition, averaged at separate 5 second intervals; C) Mean (SE) pupil dilation at fixation within the emotional areas of interest across the entire 5 second period; D) Mean (SE) pupil dilation at fixation within the emotional areas of interest, averaged across separate 5 second intervals.

Two analyses were conducted to examine condition-related differences in the proportion of dwell time within eAOIs. First, percentage dwell time was evaluated collapsing across the entire 5 second period. Repeated measures ANOVA revealed a significant effect of Condition, F (2, 48) = 18.5, p < 0.001 (partial eta squared = 0.44). As can be seen in Fig 3A, participants displayed a pattern of Distraction < Unpleasant Passive Viewing < Reappraisal (Distraction vs Unpleasant Passive Viewing: t = 3.17, p < 0.01; Reappraisal vs Unpleasant Passive: t = 3.04, p < 0.01; Reappraisal vs. Distraction: t = 5.89, p < 0.001).

Second, differences in proportion dwell time among the conditions was evaluated at each of the 5 second intervals during stimulus presentation to determine whether gaze pattern differentially shifted across time. A 3 Condition (distraction, reappraisal, unpleasant passive viewing) X 5 Time Interval (0-1s, 1-2s, 2-3s, 3-4s, 4-5s) repeated measures ANOVA revealed significant main effects of Condition, F (2, 48) = 21.6, p < 0.01 (partial eta squared = 0.47), and Time Interval, F (2.26, 54.19) = 24.33, p < 0.001 (partial eta squared = 0.50), which was qualified by a significant Condition X Time Interval interaction, F (3.66, 87.85) = 4.27, p < 0.01 (partial eta squared = 0.15) (see Fig 3B).

Follow-up repeated measures ANOVAs were conducted separately for each condition to evaluate changes in percentage dwell time within eAOIs across the 5 second period. For Distraction, proportion dwell time within eAOIs significantly changed across time, F (1.56, 37.35) = 21.8, p < 0.001 (partial eta squared = 0.48). Paired samples t-tests indicated a significant decrease in dwell time within the eAOI from seconds 1–2 (p < 0.04), 2–3 (p < 0.001), and 3–4 (p < 0.02), and a trend for 4–5 (p < 0.09). For Reappraisal, there was a significant effect of Time, F (2.04, 49.06) = 6.61, p < 0.001 (partial eta squared = 0.22). Paired-samples t-tests indicated a trend toward a significant increase in eAOI percentage dwell time from seconds 1–2 (p <0.06), and there was a significant decrease from seconds 2–3 (< 0.01) and 3–4 (p< 0.02), and a trend toward a significant decrease from seconds 4–5 (p = 0.13). For Unpleasant Passive Viewing, there was also a significant effect of time, F (2.05, 49.17) = 23.63, p < 0.001 (partial eta squared = 0.50). Paired samples t-tests indicated significant differences from seconds 1–2 and 2–3 (p’s < 0.001), but not 3–4 (p = 0.77) or 4–5 (p = 0.14). Thus, while implementing Reappraisal, participants initially showed an increase in the amount of time spent attending to emotional interest areas, which was followed by a sharp decrease in time attending to these areas starting at second 3; in contrast, during Distraction participants showed a gradual decrease in time attending to the eAOI across the 5 second trial.

### Pupil Dilation Analyses

Three sets of analyses were conducted to evaluate sympathetic nervous system activity and effortful engagement of cognitive control processes involved with emotional reactivity versus regulation. First, pupil dilation was evaluated across the entire image for the full 5 second period. Repeated measures ANOVA indicated a significant difference in pupil size across the 4 conditions, F (2, 48) = 8.36, p < 0.001 (eta squared = 0.26). Follow-up paired samples t-tests indicated that the expected emotional reactivity effect was present, where unpleasant passive viewing was greater than neutral (t = x, p < x). Emotion regulation effects were also present, such that greater pupil dilation was observed for reappraisal than distract, unpleasant passive viewing, and neutral conditions. Distract did not differ from unpleasant passive viewing, but was significantly greater than neutral.

Second, pupil dilation at fixation was evaluated within the eAOIs, collapsing across the 5-second interval. Repeated measures ANOVA indicated a significant difference in pupil size across the 3 unpleasant stimulus conditions, F (2, 48) = 6.02, p < 0.01 (eta squared = 0.20). Follow-up paired-samples t-tests indicated less pupil dilation for Distraction than Reappraisal (t = 3.3, p < 0.01), greater pupil dilation for Reappraise than Unpleasant Passive Viewing (t = 2.45, p < 0.03), and no difference between Distraction and Unpleasant Passive Viewing (t = 1.17, p = 0.25) (see Fig 3C).

Second, a 3 Condition X 5 Time Interval repeated measures ANOVA was conducted to evaluate time dependent changes in pupil dilation across conditions. The Condition X Time Interval interaction was significant, F (1.71, 40.9) = 4.96, p < 0.02 (partial eta square = 0.17), as was the main effect of Time Interval, F (1.27, 30.54) = 36.9, p < 0.001 (partial eta squared = 0.61). The main effect of Condition was nonsignificant, F (2, 48) = 2.19, p = 0.12 (partial eta square = 0.08). Follow-up repeated measures ANOVAs indicated a significant difference in pupil dilation among the 3 conditions at second 0–1, F (2, 48) = 21.04, p < 0.001 (partial eta squared = 0.47), second 1–2, F (2, 48) = 4.00, p < .03 (partial eta square = 0.14), and second 2–3, F (2, 48) = 4.67, p < 0.02 (partial eta square = 0.1g); however, the conditions did not differ at seconds 3–4, F (2, 48) = 2.2, p = 0.12 (0.08), or 4–5, F (2, 48) = 1.26, p = 0.29 (0.05). Follow-up paired samples t-tests were conducted for time intervals showing significant effects. At second 0–1, there was lower pupil dilation for Distraction than Unpleasant Passive Viewing or Reappraisal (p’s < 0.001); however, Reappraisal and Unpleasant Passive Viewing did not significantly differ (p = 0.06). At second 1–2, Distraction and Unpleasant Passive Viewing did not differ (p = 0.19); however, there was greater pupil size for Reappraisal than Distraction and Unpleasant Passive Viewing (p’s < 0.05). At second 2–3, Reappraisal was significantly higher than Unpleasant Passive Viewing (p < 0.04); however, there was no difference between Reappraisal and Distraction (p = 0.25) or Distraction and Unpleasant Passive Viewing (p = 0.10).

Follow-up repeated measures ANOVAs were also conducted separately for each condition to evaluate changes in pupil dilation across the 5-second period. For Distraction, there was a significant effect of Time Interval, F (1.75, 41.98) = 30.77, p < 0.001 (partial eta-square = 0.56). Paired samples t-tests indicated a significant increase in pupil dilation from seconds 1–2, 2–3, and 3–4 (all p’s < 0.001), but not seconds 4–5 (p = 0.64). For Reappraisal there was a significant effect of Time Interval, F (1.17, 28.13) = 18.79, p < 0.001 (0.44). Paired samples t-tests indicated significant increases in pupil dilation from second 1–2, 2–3, and 4–5 (p’s < 0.001); there was no difference between seconds 3–4 (p = 0.64). For Unpleasant Passive Viewing, the effect of Time Interval was also significant, F (1.41, 33.7) = 37.22, p < 0.001 (0.66). Paired samples t-tests indicated a significant increase in pupil dilation from seconds 1–2, 2–3, 3–4, and 4–5 (p’s < 0.001).

As can be seen in Fig 3D, pupil dilation generally increased in all 3 conditions over the course of the 5 second trial. However, there were important differences among conditions. During the first second of the trial, Distraction resulted in less pupil dilation than Reappraisal or Unpleasant Passive Viewing conditions; however, over the subsequent 4 seconds, Distraction and Unpleasant Passive Viewing had more comparable levels of pupil dilation. In contrast, reappraisal showed a steeper increase in pupil size over the 5 second epoch, which was greater in magnitude than both Distraction and Unpleasant Passive Viewing from second 2 onward.

### Correlations

Bivariate correlations were calculated to determine whether self-reported emotional experience is associated with proportion dwell time within E-AOIs and pupil response. To isolate regulation effects within the behavioral data, a difference score was calculated as regulation condition–unpleasant passive viewing. Higher difference scores reflect poorer emotion regulation. Proportion dwell time within the E-AOI was evaluated in early and late trial epochs: early = average of 0–1 and 1–2 seconds, late = average of 2–3, 3–4, and 4–5 seconds. A significant positive correlation was observed between the behavioral ratings for Reappraisal and proportion dwell time within the E-AOI for Reappraisal during late (r = 0.42, p < 0.04), but not early (r = 0.12, p = 0.57) epochs. Similarly, a significant positive correlation between behavioral ratings and E-AOI dwell time was observed for Distraction during the late (r = 0.40, p < 0.05), but not early (r = 0.35, p = 0.09), trial epoch. Self-reported negative emotion was not significantly associated with pupil dilation during early or late trial epochs. Thus, poorer emotion regulation during Distraction and Reappraisal was associated with greater proportion of dwell time within E-AOIs during later portions of the trial.

## Discussion

The current study used eye tracking and pupil dilation to evaluate whether reappraisal and distraction emotion regulation strategies are associated with distinct patterns of visual attention and differences in the extent to which they recruit effortful cognitive control processes. Several important findings emerged. First, reappraisal and distraction were both effective at reducing the subjective experience of negative emotion. These findings replicate and extend prior work using Event Related Potentials (ERPs) which found both of these strategies to significantly reduce the amplitude of the Late Positive Potential (LPP), an ERP component associated with emotion regulation success [20]. Importantly, behavioral ratings in these conditions did not differ, suggesting that they may be equally effective at reducing negative affect. Second, although behavioral ratings did not differ between reappraisal and distraction, these conditions achieved their regulatory effects through very different patterns of visual attention. Specifically, distraction was associated with decreased attention to arousing scene regions even by the first second and showed further decreases in attention to arousing interest areas as the trial went on. While implementing distraction, participants also had fewer total fixations across the entire image. When viewed together, these two results suggest that effective implementation of distraction requires rapidly orienting attention away from aversive content and then leaving gaze relatively stable within a less arousing scene region while implementing the strategy. Reduced total visual scanning may be necessary during distraction due to the cognitive demand associated with generating mental representations of neutral content; this process may tax working memory capacity to an extent that visual scanning of external cues is less feasible. In contrast, reappraisal resulted in an initial increase in dwell time to arousing scene regions, followed by a shift in attention toward less arousing scene regions. When viewed in conjunction with other published studies on reappraisal, it is likely that effective reappraisal requires: 1) bottom-up attention to be initially oriented toward arousing content, followed by purposeful allocation of top-down attention to arousing scene regions to appraise stimulus valence and motivational relevance; 2) shifting attention toward less arousing scene regions, which may facilitate the inhibition of the initial prepotent appraisal of the stimulus; and 3) the retrieval of information from semantic memory that can be used to intentionally select a new stimulus-appropriate appraisal that is consistent with regulatory goals. Our reappraisal findings are generally consistent with those reported by prior studies indicating that individuals are less likely to fixate on emotional interest areas while applying reappraisal; however, our results provide greater temporal specificity than past studies which collapsed epochs into broader intervals (e.g., early 1-3S; middle 4–6; late 7–10) which may have missed valuable information regarding early increases in attention to arousing interest areas (e.g., [9]). Eye tracking differences observed between our two regulation conditions may explain why Thirukselvam et al. [20] observed that distraction resulted in a more rapid decrease in amplitude of the LPP than reappraisal. Specifically, participants may attend to less arousing scene regions at an earlier time point during distraction, which is not possible during reappraisal because early attentional orienting is needed to generate an initial appraisal that can be subsequently replaced by a goal-relevant reappraisal. Furthermore, although the current findings suggest that attention plays an important role in effective reappraisal and distraction during natural (i.e., unconstrained) conditions, studies requiring participants to reappraise unpleasant stimuli while holding visual attention constant make it clear that effective reappraisal can be accomplished without shifting attention away from arousing content [22, 23]. We suspect that this is also true of distraction and that healthy individuals with adequate working memory capacity should be able to generate a mental representation of content other than the presented stimulus that is powerful enough to reduce negative affect, even when visual attention is held constant on arousing interest areas. Future studies are needed to test this possibility.

A third major finding was that better subjective emotion regulation was associated with decreased dwell time to arousing interest areas during later trial epochs for both reappraisal and distraction. Individual differences in the ability to disengage attention from arousing content and shift toward less arousing scene regions may therefore be an important predictor of emotion regulation effectiveness. Our results are consistent with those of van Reekum et al. [8] and Urry et al. [17] who found that attending to less arousing interest areas was associated with reductions in amygdala response while participants decreased negative emotion using reappraisal. These findings contradict those of Bebko et al. [9]who found that better emotion regulation was associated with *greater* probability of fixating on arousing interest areas. Discrepant results across these two studies may reflect methodological factors, such as stimulus presentation duration (5 seconds in the current study vs 10 seconds in [9]). Longer presentation times may allow additional cognitive processes to be implemented during reappraisal compared to shorter presentation times. For example, Ochsner et al. [18] proposed that conflict monitoring is a critical component of reappraisal, i.e., the ability to track the extent to which a current reappraisal is changing emotional response in conjunction with regulatory goals. In tasks using very long stimulus durations (e.g., >10s), participants may have enough time to adequately engage in conflict monitoring and potentially start the reappraisal process over again if they are not meeting their regulatory goals. Such instances would invariably require greater allocation of attention to arousing interest areas in an effort to generate more effective reappraisals on the second pass attempt. Although speculative, it is possible that the seemingly paradoxical correlation observed in Bebko et al. [9] (i.e., better emotion regulation being associated with greater attention to arousing interest areas) is informative, suggesting that individuals who engage in conflict monitoring may have better emotion regulation outcomes, in part because they are able to use attention to adaptively restart the reappraisal process after initial failures.

A fourth major finding was evidence for differences in pupil dilation across the conditions, such that reappraisal was associated with greater increases in pupil size compared to unpleasant passive viewing and distraction conditions. It is well-established that greater pupil dilation is associated with increased emotional arousal and sympathetic nervous system activity [11], as well as increases in cognitive control and the recruitment of effortful cognitive processes [12, 14–16]. Emotion regulation studies have used pupil dilation as an index of the recruitment of effortful cognitive control processes and found that increasing or decreasing negative affect via reappraisal results in greater pupil size than passively viewing unpleasant stimuli [8, 17]. Our results are consistent with these prior findings on reappraisal, and extend them by indicating that distraction did not result in comparable increases in pupil size; rather, pupil dilation during distraction was similar to unpleasant passive viewing. Although reappraisal and distraction may be relatively equivalent in their effectiveness at decreasing negative affect, distraction may be a less effortful and less cognitively demanding emotion regulation strategy. The neural basis for how attention, emotion, and control related emotion regulation processes interact requires further exploration. The amygdala provides both direct and indirect top-down signals to sensory pathways, producing modulatory effects that might supplement, but also compete with other sources of top-down control (e.g., emotion regulation) [24]. It is therefore possible that the differences we observed between reappraisal and distraction conditions reflect complex modulatory effects and interactions between multiple sources of top-down control (i.e., those that are attention versus emotion regulation related).

The current findings have implications for theoretical frameworks on emotion regulation and the development of novel interventions. The process model of emotion regulation [3] proposes that strategies can be implemented to affect the four stages of the emotion generation sequence: stimulus, attention, appraisal, response. This assumes that strategies affecting these stages operate at least to some extent in isolation. However, the current findings suggest that there may be complex interactions among these stages; we suspect that attention may play an important role in decreasing negative affect at each of the 4 stages of the emotion generation sequence. The current results demonstrate how bottom-up and top-down attention are both critically involved in effective reappraisal, operating both prior to and during the reappraisal process. Attention likely plays a similar role in other strategies (e.g., situation selection, situation modification, and affective suppression), both determining which stimuli are gated into working memory and subjected to more elaborative processing and by facilitating the execution of the strategy itself. Notably, these processes may operate at both automatic and controlled levels of processing [4] and individual differences in bottom-up and top-down emotion-attention interactions while individuals are implementing these strategies may determine how effectively they can reduce negative affect. It is possible that greater propensity toward having bottom-up attention captured by unpleasant stimuli or greater difficulty disengaging top-down attention from unpleasant stimuli plays a critical role in emotion regulation abnormalities that occur in several forms of psychopathology. Translational research that uses eye tracking to isolate different components of attention while clinical and healthy comparison groups attempt to decrease negative affect using various strategies is warranted to evaluate this possibility. Such studies may be particularly relevant for psychiatric (e.g., schizophrenia; [25, 26]) and neurological disorders (e.g., hemispatial neglect; [27, 28]), for whom the coupling of peripheral arousal and central nervous system activity may be heightened or reduced as a function of attentional processes.

Certain limitations should be considered. First, the emotion regulation instructions may be prone to demand characteristics. Asking participants to feel more neutral may artificially drive a lot of the decrease in subjective negative emotion, with demand characteristics playing a greater role than the strategy per se. Second, the implications of the distraction findings are to be determined. Some therapies and theories discourage the use of distraction because it limits full emotion processing for long-term reduction of negative emotion. This may cause unwanted effects if individuals are re-exposed to unpleasant stimuli at a later time. For example, using ERPs Thiruchselvam et al. [20] showed that that distraction lead to a rebound in emotional reactivity upon re-exposure to the pictures. Third, our subjective ratings of negative emotion were only taken after the emotion regulation strategy was implemented. This makes it difficult to know whether there was a change in experience or if participants simply found the stimulus less negative to begin with because a separate rating of the stimulus prior to applying the strategy was no obtained. Fourth, stimuli representing different discrete negative emotions show a moderate degree of temporal coherence between subjective and neurophysiological responses, such that both phenomenon tend to unfold on similar time courses [29]. Recent evidence suggests that emotion regulation strategies may disrupt this temporal coherence between physiological and subjective responses [30]; however, some strategies are more prone to disrupt coherence than others (e.g., suppression > acceptance). It is unclear whether successful emotion regulation requires disruption of the coherence of emotion responses across subjective, physiological, and behavioral domains or if some strategies are more effective than others because they cause such disruption. We suspect that attention may be a key moderator of whether coherence is disrupted and different strategies are effective. Given that the current results demonstrate that different strategies are associated with distinct patterns of visual attention, this represents an important future direction.

Despite these limitations, results suggest that it may be beneficial to incorporate attention training procedures into emotion regulation focused interventions. Several psychosocial interventions have been developed to target emotion regulation abnormalities [31]. These interventions typically include elements of emotional awareness and learning to control attention towards both internal mental representations and external cues. For example, clients are taught to attend to internal states by participating in activities such as breathing exercises, as well as reflecting on situations that elicit conflicting emotions and spending time defining and separating them. Skills are also taught to direct attention toward external sensory experiences (e.g., taste) to increase attentional flexibility. The current results suggest that it may also be beneficial to develop computerized attention training programs to accompany these psychosocial interventions, which could systematically shape clients with emotion regulation deficits to attend to external cues in a manner similar to individuals who are more effective at regulating their emotions. Attention Bias Modification Training (ABMT) programs have been developed for the purpose of reducing clinical symptoms associated with psychiatric conditions, and found to be efficacious after clients have completed multiple weekly sessions [32]. Components of ABMT could be adapted and tailored to the purposes of shaping attention to what is most effective during specific emotion regulation strategies. Similar to how cognitive remediation programs have been developed and found effective for improving general memory and executive functioning, it may be beneficial to develop emotion regulation focused cognitive rehabilitation programs. Finally, our pupil dilation results suggest that some strategies (e.g., reappraisal) are more cognitively effortful than others, even though different strategies may result in similar decreases in negative emotion. This knowledge may be highly beneficial for treating psychiatric disorders that exhibit both cognitive impairment and emotion regulation abnormalities (e.g., schizophrenia) [25, 26]. In such disorders, it may be more beneficial for clinicians to help patients develop skills in applying less cognitively demanding strategies, such as distraction, that prove equally effective at reducing negative affect but are less effortful. Adaptively selecting emotion regulation strategies to implement based upon cognitive ability and willingness to engage in effortful cognitive processes may be a necessary ingredient in successful emotion regulation treatment.

## Appendix

Neutral Images:

2190, 2221, 2235, 2280, 2320, 2383, 2393, 2398, 2440, 2480, 2495, 2516, 2560, 2579, 2580, 2749, 2840, 2850, 2870, 7025, 7090, 7175, 7211, 7217, 7493, 7496, 7550, 9210

Negative Passive Viewing Images:

1050, 1111, 1300, 1930, 2700, 3030, 3053, 3059, 3060, 3168, 3181, 3213, 3220, 3230, 3300, 3301, 3350, 6415, 6830, 9006, 9040, 9042, 9181, 9433, 9570, 9571, 9800, 9810

Distraction Images:

1201, 2095, 2141, 2399, 2710, 2717, 2750, 2800, 3064, 3100, 3101, 3140, 3550, 6211, 6313, 6350, 6360, 6510, 6530, 6550, 6555, 9252, 9253, 9300, 9301, 9420, 9635.1, 9921

Reappraisal Images:

2120, 2130, 2375.1, 2683, 2691, 2810, 2900.1, 2981, 3000, 3010, 3015, 3120, 3130, 3160, 3400, 3530, 6212, 6230, 6243, 6570, 6821, 6831, 7359, 7380, 9050, 9265, 9405, 9410

## Supporting Information

S1 Data S1 Data includes the file all analyses were conducted on. (SAV)
